# CAR T cell-based immunotherapy and radiation therapy: potential, promises and risks

**DOI:** 10.1186/s12943-023-01775-1

**Published:** 2023-05-12

**Authors:** Lusine Hovhannisyan, Carsten Riether, Daniel M. Aebersold, Michaela Medová, Yitzhak Zimmer

**Affiliations:** 1 Department of Radiation Oncology, Inselspital, Bern University Hospital, Freiburgstrasse 8, Bern, 3008 Switzerland; 2 Department for Biomedical Research, Radiation Oncology, University of Bern, Murtenstrasse 35, Bern, 3008 Switzerland; 3 Graduate School for Cellular and Biomedical Sciences, University of Bern, Bern, 3010 Switzerland; 4 Department of Medical Oncology, Inselspital, University Hospital and University of Bern, Bern, 3010 Switzerland

**Keywords:** Radiation therapy, CAR T cell therapy, Immunotherapy

## Abstract

CAR T cell-based therapies have revolutionized the treatment of hematological malignancies such as leukemia and lymphoma within the last years. In contrast to the success in hematological cancers, the treatment of solid tumors with CAR T cells is still a major challenge in the field and attempts to overcome these hurdles have not been successful yet. Radiation therapy is used for management of various malignancies for decades and its therapeutic role ranges from local therapy to a priming agent in cancer immunotherapy. Combinations of radiation with immune checkpoint inhibitors have already proven successful in clinical trials. Therefore, a combination of radiation therapy may have the potential to overcome the current limitations of CAR T cell therapy in solid tumor entities. So far, only limited research was conducted in the area of CAR T cells and radiation. In this review we will discuss the potential and risks of such a combination in the treatment of cancer patients.

## Introduction

Cellular therapies based on the transfer of immune cells have entered clinical practice as “living drugs”. These modified immune cells may have therapeutic potential not only for oncological indications but also for inflammatory and infectious diseases [1]. Patient T cells being genetically modified to recognize surface molecules on tumor cells using a chimeric antigen receptor (CAR), consisting of an antigen recognition domain based on an antibody single-chain variable (scFv) domain or receptor-ligand-based domain [2], represent the lighthouse development in the field and a tremendous success in the treatment of hematological malignancies [3]. Within the last few years, CAR T cell therapies have been used for management of hematological malignancies, such as refractory or relapsed acute lymphoblastic leukemia (r/r B-ALL), diffuse large B-cell lymphoma (DLBCL), mantle cell lymphoma (MCL) and more recently multiple myeloma. Currently, great efforts are undertaken to broaden the application for solid tumor types and non-malignant diseases [4–6]. However, present limitations of CAR T cell therapy in solid tumor entities such as reduced homing to the tumor, an immunosuppressive tumor microenvironment, heterogeneous expression of tumor antigen, limited vascularization and hypoxia have not been tackled successfully yet [7]. Immune checkpoint inhibitor (ICI) therapy is active in many solid tumor entities with its efficacy being limited through similar mechanisms as identified for CAR T cell therapy [8]. Some of these limitations can be overcome by combining ICI therapy with radiation [9], identifying radiation therapy (RT) as attractive combination partner for CAR T cell therapy.

RT is a classical long-established tumor treatment modality used for more than half of cancer patients in different sequences and combination regimens. Recent discoveries point toward a profound impact of RT on the tumor microenvironment (TME), activation of the immune response, induction of neoantigen expression, and systemic immunomodulatory effects [10]. RT disturbs mechanical, functional as well as dynamic barriers on the way of T cells to enter the tumor [11]. RT induces DNA damage and thus promotes the occurrence of new mutations within the tumor. These *de novo* mutations often result in the expression of cancer antigens and neoantigens, hence favoring CAR T cell therapy initiation after RT when CAR target antigen expression is induced [12–15].

The overall impact of RT on (CAR) T cells can be classified either as target antigen-independent or target antigen-specific effects. Whereas antigen-independent mechanisms are general and alter for example the expression of IC molecules, antigen-specific mechanisms include induction of CAR target antigens by increasing their expression on tumor surface, or by favoring the survival of antigen-positive cells [16].

Inhibition of antitumor immunity via checkpoint molecules is one of the key resistance mechanisms in solid tumors [17]. This can also hinder the response to CAR T cell therapy. To overcome CAR T cells exhaustion, a favorable combination with RT could alleviate the anti-tumor activity and persistence of the immune cells [18].

**Fig. 1:**
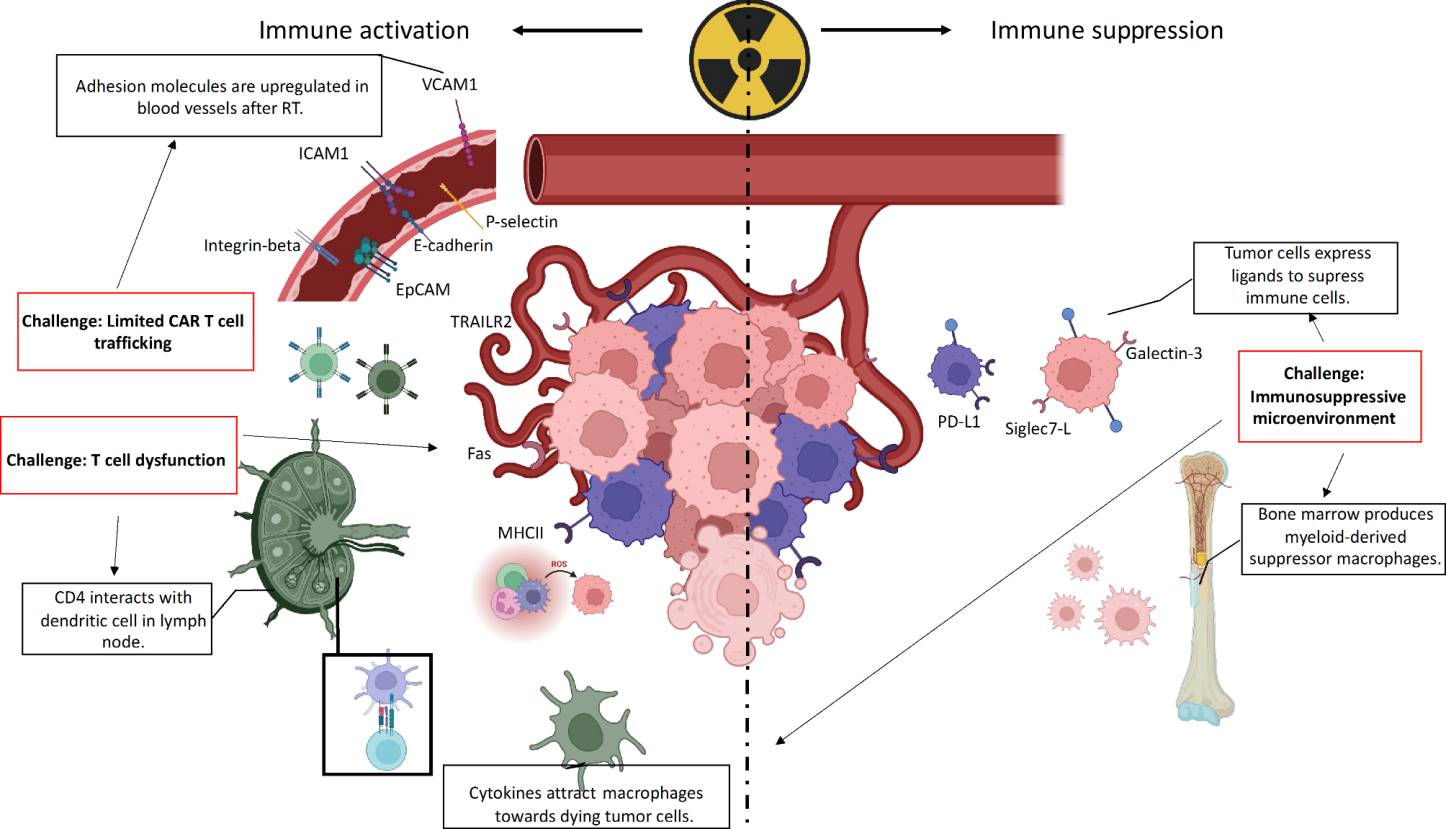
Radiation effects on immune system. *Left*: Immune activation effects include upregulation of adhesion molecules that enhance immune infiltration, increased antigen expression, cytokines and reactive oxygen species generation, macrophages chemoattraction, with further antigen presentation in lymph nodes and T cell priming. *Right*: Immune suppression. Chemokines stimulate bone marrow to produce myeloid-derived suppressor cells. Tumor cells maintain or overexpress ligands for T cells that will suppress them and create immunosuppressive microenvironment. Challenges that affect these mechanisms are highlighted (*red rectangles*) and connected to the processes affected by RT (*dotted arrows*). (Created with BioRender.com.)

Considering the remarkable progress in the development of CAR T cell therapy in recent years as well as the growing understanding of the RT-mediated immune effects (Fig. 1), this review focuses on the biological effects of both these therapies and highlights potential benefits and limitations of their combination as part of the next generation cancer treatment.

## Immunological effects of RT

Although RT has been traditionally considered a local therapy, it became clear that this treatment induces various biological effects also on non-irradiated tissues. These effects are classified as either ‘bystander’ or ‘abscopal’, referring to the ‘neighboring’ or ‘far-away’ events, respectively [19]. The ‘bystander’ effect is resulting from intercellular crosstalk mediated by gap junctions and through the release and transfer of soluble factors such as cytokines or reactive oxygen/nitrogen species whereas the ‘abscopal’ effect is possibly mediated through immune cells and cytokines circulating in the blood [20].

RT-induced cell death is classically attributed to generation of free radicals, unrepaired DNA damage, and accumulation of damaged macromolecules [21]. Recently, RT-induced immune-mediated cell death became a topic of investigation. RT can overcome barriers caused by the immunosuppressive tumor microenvironment, increase antigen priming of T cells and immune cell infiltration, as well as antigen presentation thus inhibiting the immune escape [22].

Within the idea of combining RT with immunotherapy (IMT), different RT doses and delivery schedules were proposed due to the very different biological effects. While higher RT doses induce necrosis and massive inflammation, optimally timed lower doses are less immunosuppressive [23].

RT activates expression of damage-associated molecular patterns (DAMPs) such as high mobility group box 1 (HMGB1). Dendritic cells bind to HMGB1 and activate Toll-like receptor 4-dependent pathways [24, 25]. RT also induces expression of adhesion molecules for immune cells including intercellular adhesion molecule 1 (ICAM1) and vascular cell adhesion molecule 1 (VCAM1) [26]. Effect of RT on upregulation of VCAM1 affecting the hematopoietic-stem cell rolling was demonstrated on irradiated rat fetuses [27].

RT affects tumor infiltration of immune cells. A single dose of 10 Gy of neoadjuvant RT showed positive effect on the infiltration of natural killer (NK) cells when combined with the antitumor immunocytokine-based drug L19-IL2 [28]. Another study showed that 3 fractions of 20 Gy act synergistically with immunostimulatory treatment by the OX40 (immunostimulatory checkpoint expressed on T cells) agonist antibody in a murine sarcoma model in T cell-dependent manner [29].

Deng et al. described a mechanism that can be responsible for an improved adaptive immune response to RT and involves the cytosolic DNA sensor cyclic GMP-AMP synthase-Stimulator of Interferon Genes (cGAS-STING) pathway, which activates interferon regulatory factor 3, leading to type I interferon (IFN-I) production. The importance of STING in this cascade was demonstrated in an in vivo model comparing STING-wild type to STING-deficient mice. In the lymph nodes of STING-deficient animals, significantly fewer antigen-specific CD8 + T cells were found in response to tumor irradiation [30]. This observation can be explained by RT-based activation of STING, which in turn activates IFN-I and nuclear factor ‘kappa-light-chain-enhancer’ of activated B-cells (-κB)(NF-κB) pathways, resulting in the production of interferon β (IFN-β) and other pro-inflammatory cytokines.

One of the recent advances in clinical application of the immune stimulatory role of RT was demonstrated in a proof-of-principle clinical trial NCT02474186 assessing abscopal effects. The trial enrolled patients with various malignancies, including non-small cell lung, breast, thymic, cervical and ovarian cancer. The regimen consisted of two cycles of RT of 35 Gy in 10 fractions within two weeks, each followed by two weeks administration of granulocyte-macrophage colony-stimulating factor [31]. Around 27% of abscopal responses (defined as at least 30% volume decrease of non-irradiated lesion) were achieved in patients treated with RT combined with immune stimulation. These results indicate the capability of RT to induce patients’ immune response in different tumor types when given in a neoadjuvant setting before immune stimulation.

Apart from immune activation, RT can also induce immune suppression, as bone marrow and lymph nodes are among the most radiosensitive tissues [32, 33]. In addition, RT can cause chronic inflammation, which favors immune suppression with the involvement of immune cells such as myeloid-derived suppressor cells and tumor-associated macrophages [34].

Although only a few studies are being conducted in the field of adoptive T cell therapies, there is preclinical and clinical data demonstrating that protocols where RT precedes adoptive T cell therapy yield better response and better infiltration with T cells compared with protocols not involving RT. This was shown in various tumor types including melanoma, gastric cancer, pancreatic cancer, and multiple myeloma [35].

## Immune checkpoints and RT

Immune checkpoints (ICs) are inhibitory receptors on immune cells and their ligands are usually upregulated in cancer. Known ICs expressed on T cells are cytotoxic T-lymphocyte-associated protein 4 (CTLA4), programmed cell death protein 1 (PD-1), lymphocyte-activation gene 3 (LAG3), T-cell immunoglobulin and mucin-domain containing-3 (TIM-3), T cell immunoglobulin and ITIM domain (TIGIT), B- and T-lymphocyte attenuator (BTLA), and Sialic acid-binding immunoglobulin-like lectin (Siglec) receptors [36, 37]. They are therapeutically targeted via blocking antibodies, and those therapies showed promise in combination with RT [38, 39]. As CAR T cells are modified T cells, they express ICs and may be affected by RT-induced IC receptor/ligand modulations [36, 40]. There is evidence supporting CAR T inhibition upon interactions with IC ligands, and several strategies are implemented to reduce this effect [40]. Combining CAR T and IC blockade was successful in preclinical studies, and currently is tested in early clinical trials [40]. We further discuss how RT impacts IC modulation (Table 1), and how this may influence RT and CAR T cell therapy combination.

CTLA4/CD80 can be potentiated by upregulation of CD80 mediated via TNF-alpha as observed in A20-HL B lymphoma cell line within 12 h post 8 Gy of irradiation [41, 42]. CTLA4 blocking and RT combination in metastatic solid cancer patients correlated with increased CD8 + T responses in peripheral blood, and tumor reduction outside the RT field [43, 44].

PD-1 interaction with its ligand PD-L1 was induced after RT in many cancer types. PD-L1 expression was upregulated in bladder cancer cell lines in a RT dose-dependent manner following up to 9 Gy, with the highest in vitro effect observed 48 h post irradiation [45]. Similar response was documented in vitro in the non-small cell lung cancer (NSCLC) cell line A549, where PD-L1 expression was upregulated in a time- and dose-dependent manner after treatment of up to four 6 Gy fractions [46]. In vivo, mice bearing syngeneic tumors subjected to 12 Gy of RT had a peak of PD-L1 expression at 72 h post irradiation, which was significantly reduced after 7 days [45]. Similarly, GL261-luc2 glioma cell line increased PD-L1 expression after two doses of 5 Gy and five doses of 2 Gy [47].

TIM-3 is a receptor with several soluble ligands excreted into the microenvironment by dying tumor cells [48]. These ligands include HMGB1, carcinoembryonic antigen cell adhesion molecule 1 (Ceacam-1), and a surface molecule phosphatidylserine [49–52] and they all have been shown to increase after RT in various cancers [53–55].

LAG3/GAL-3 interaction is another inhibitory mechanism for T cells [56, 57]. Overexpression of LAG3 as well as of other checkpoint players such as PD-L1 and TIM3, was detected in the microenvironment of irradiated murine esophageal cancer [58]. GAL-3 expression increased also in the U87MG glioma cell line 72 h after exposure to 0.5-12 Gy RT combined with temozolomide in vitro [59, 60]. In the 5T33 murine myeloma model, 5 Gy of whole body irradiation (WBI) resulted in increased LAG3 and TIM3 expression on T cells assessed 12 days after treatment [61]. Additionally, these tumors responded well to RT in combination with one or more checkpoint inhibitors resulting in a synergistic effect and increased the percentage of cured animals. The best outcomes following WBI were observed upon combination treatments targeting together either PD-L1 and LAG3, PD-L1 and TIM3, or PD-L1 and CTLA-4 [61]. Another approach combining 10 Gy RT, anti-PD-L1, and anti-TIM-3 antibodies resulted in a 100% long-term survival of GL261-luc2 murine glioma-bearing mice [62].

Interestingly, when RT led to a higher immune stimulation due to damage and inflammation by employing high dose fractions, it also induced higher expression of inhibitory molecules. In a study where mice implanted with murine colon cancer CT26 cell line received 3 different RT schemes (1*16.4 Gy, 3*8Gy, or 18*2Gy), the 3*8Gy scheme induced the highest T cell response but at the same time increased TIGIT expression [63].

To summarize, IC molecules inhibit activation of T cells, including CAR T cells, and this effect can be targeted via checkpoint blockade. Recent research identified that checkpoint molecules are modulated by RT and this modulation is affected by RT timing and dosage. Thus, the mechanisms behind these effects need to be considered when treatment schedules are being adapted, for example to prevent CAR T inhibition via RT-induced IC expression.

**Table 1:** Examples of antigens that are used as targets in particular cancer cells and their response to RT. Increase in antigen levels post RT follows with distinct timing and the effect lasts for several days up to one week

Tumor type / model	Radiation regimen	Antigen	Effect	Reference
CT26 murine colon carcinoma cell line (in vitro)	75 Gy	Phosphatidylserine	Increase to 4% at 4 h and 10% at 24 h	Obeid et al., 2007 [52]
HT-29 human colon adenocarcinoma cell line (in vitro)	8 Gy*1, 2 Gy*4	Various (SILAC-based proteomic study after RT)	KPNA2, MART3, LCN2, OAT, TK1, HSPBL1, KRT20, KRT19 upregulated (expression assessed at 24 h, 48 h, 72 h)	Song et al., 2016 [64]
Burkitt lymphoma cell lines Daudi and Raji (in vitro)	0.5–2.0 Gy	CD20	Increased expression within 24 h	Gupta et al., 2008 [65]
Neoplastic B-cell lines (IM9, IM9/Bcl2, Ramos) (in vitro)	5,10,15,20, 20 Gy	CD20	Dose-dependent upregulation up to 10 Gy, peaking at 24 h and disappearing at 48 h	Kunala and Macklis, 2001 [66]
HT-29 human colon adenocarcinoma cell line (in vitro)	6 Gy	MUC1	Two-fold increase from day 1 after RT, continuation of increase up to day 4	Kang et al., 2000 [67]
MKN45 gastric adenocarcinoma cell line (in vitro)	5-15 Gy	CEA	Increased surface expression 4 days after RT in a dose-dependent manner	Hareyama et al., 1988 [68]
CT26 murine colon carcinoma cell line (in vivo)	2 Gy*5	PD-L1	Increased expression within one week, effect away after day 7	Dovedi et al., 2014 [69]

## Combining radiotherapy and CAR T cells in solid tumors

### Challenges

The obstacles for CAR T cell therapy success (Table 2) can be classified as either tumor microenvironment (TME)-related or target antigen-dependent. TME-related challenges include limited T cell homing to the tumor, an immunosuppressive TME, limited vascularization and hypoxia [70, 71]. Target antigen-related obstacles for CAR T cell therapy success are associated with a poor and heterogeneous expression of targeted tumor antigen on cancer cells. Tumor cells need to express at least 1000 tumor antigens on the cell surface to be recognized by CAR T cells [72]. Furthermore, acquired resistance to CAR T cell therapy is mediated by downregulation or loss of target antigen [70, 71].

The TME is a crucial tissue compartment through nurturing and protecting cancer cells from the immune response. The TME consists of an extracellular matrix (ECM) and non-malignant cells such as vascular endothelial cells, fibroblasts, tumor-associated macrophages (TAMs), and T cells [73]. ECM and fibroblasts surround neoplastic cells, creating a physical barrier on the way of CAR T cells that cannot exhibit their cytotoxic function without direct contact with tumor cells. Moreover, CAR T cells encounter hypoxic conditions, acidic pH, and aberrant vasculature that restricts trafficking and potential necessary surviving physiological conditions that hamper their cytotoxic activity within the tumor TME [70, 73]. Additional challenges are imposed by regulatory T cells (Tregs), myeloid-derived suppressor cells, and M2 macrophages that decrease the potency of effector T cells [73]. Hypoxic conditions exclude effector T cells and create favorable conditions to recruit myeloid-derived suppressor cells [74]. In addition, TME cells have an imbalance in the production of chemokines in favor of immune inhibition. They express fewer immune stimulatory receptors CXCR3 and CCR5 and produce more suppressor ligands CCL17/22 and CCL2 [70]. The TME does not only restrict CAR T cell trafficking but also exhausts the cells infiltrating the tumor despite the abovementioned challenges. The exhaustion is mediated via myeloid-derived suppressor cells-secreted TGF-beta and IL-10, as well as M2-macrophage-secreted IDO (indoleamine 2,3-dioxygenase) [75][76]․ TAMs also mediate PD-L1 expression on tumor cells via secretion of IFN-gamma. TAM-released IFN-gamma activates JAK/STAT3 and PI3K/AKT in tumor cells, leading to PD-L1 overexpression, as demonstrated for example in a lung cancer model [77]. Another mechanism of T cell suppression is the accumulation of the byproducts of pathological metabolism, such as purines and ATP [78].

Tumor heterogeneity and antigen escape are significant hurdles for CAR T cell-based therapy, and CAR target antigen selection is challenging. Tumor bulk comprises of heterogeneous cells expressing various antigens, having different proliferation potentials and sensitivity to given treatments [79]. An optimal antigen candidate should be constantly expressed in all tumor cells and be absent in normal tissues. As such scenario seems to be elusive, current CAR T cell-based approaches aim to target the majority of tumor cells and to escape regular tissue binding and toxicity at therapeutic doses [79, 80]. However, even low expression in normal tissues can have dramatic consequences. A case report of a patient with metastatic colon cancer described acute respiratory distress 15 min upon administration of ERBB2-targeted CAR T cells. Lung infiltration occurred possibly due to expression of ERBB2, that led to CAR T cells accumulation, cytokine storm (increase in IL-6, TNF-alpha, and IFN-gamma plasma levels), and eventually to patient’s death 5 days after CAR T cells injection [81]. In this context, murine models have been ineffective in predicting target toxicities, and novel improved approaches are urgently needed. To improve the control of CAR T cell activity in the case of such events, logic gating (activation of CAR T cells via recognition of two tumor antigens) and the employment of suicide genes (drug inducible CAR T cell death program) were proposed [82].

### Rationale for combining RT and CAR T cell-based therapies

#### Impact of RT on TME

RT may be able to address some of the challenges imposed by the physiology and composition of the TME via direct and indirect impacts on its components. RT regulates the ECM via increasing its permeability and improving penetration of T cells. It creates favorable conditions for T cells’ extravasation via upregulating integrins ICAM-1 and VCAM-1 [83]. Moreover, RT may alter the tumor vasculature and prevent the immunosuppressive effects of hypoxia. For example, it has been demonstrated that radiation modulates the expression of adhesion molecules on endothelial cells after conditioning RT in a pancreatic cancer model, facilitating T cell infiltration [84]. Furthermore, neoadjuvant local low-dose gamma irradiation has been suggested to reverse tumor aberrant vasculature and efficient recruitment of tumor-specific T cells in human pancreatic carcinomas through re-programing of tumor-associated macrophages [85]. In addition, RT may modulate local expression of chemokines such as the C-C motif chemokine ligand 10 (CXCL10), CXCL16, CCL5 that can chemoattract T cells, including CAR T cells, and facilitate their penetration into the tumor [71]. Low doses of irradiation may regulate tumor vasculature and thus may improve T cell infiltration to the tumor site. In this sense, Murty et al. demonstrated improved infiltration of intravenously injected CAR T cells into a mouse brain tumor model [86]. However, the ablative doses of irradiation might induce endothelial cell death and may lead to regions of hypoxia that can potentially impair T cell-mediated antitumor effects [87].

#### Tumor antigen expression

In a 2018 manuscript by DeSelm et al., RT has been proposed to trigger apoptosis and overcome antigen heterogeneity issue by influencing the TRAIL pathway. In that respect, the authors demonstrated the impact of low dose 2 Gy radiation on CAR T cell-mediated killing in a mouse model of pancreatic cancer. This model comprised heterogenous population of sialyl Lewis-A (sLeA) antigen-positive and -negative tumor cells. Consequently, the introduced CAR T cells efficiently eliminated pre-irradiated sLeA-positive cells, upregulated TRAIL, and targeted sLeA-negative cells via a TRAIL-mediated mechanism [88].

RT can also induce target antigen expression as demonstrated in a murine model of glioma [89, 90]. The NKG2D ligands are associated with cellular stress and boosted upon cytotoxic therapies such as RT or chemotherapy. NKG2D-targeted CAR T cells exerted synergistic response combined with a single dose of 4 Gy irradiation via increased antigen expression, release of cytokines, and improved penetration into the brain. The treatment response was durable, CAR T cells efficiently proliferated and prevented tumor rechallenge [89].

#### T cells and RT

Already low doses of RT have deleterious effects on lymphocytes in lymph nodes. However, tumor resident T cells have a more radioresistant phenotype [91]. Arina et al. demonstrated that tumor-residing and antigen-experienced T cells are more radioresistant than naïve T cells or memory T cells in the spleen. Intratumoral T cells not only survived RT but also increased production of IFN-gamma and contributed to tumor control [91]. These findings indicate the potential of tumor resident CAR T cells to survive RT and have a longer-lasting antitumor capacity.

Interestingly, a subset of T cells that expresses gamma and delta T cell receptors (TCR), namely gamma/delta T cells, is considered a bridge between innate and adaptive immune response [92]. Willcox et al. described gamma/delta T cells as ‘natural’ CAR T cells due to their ability to recognize non-MHC proteins [93]. Specific populations of gamma/delta cells can tolerate therapeutic RT doses or can be modified to be resistant to cytotoxic chemotherapy [94, 95]. Those characteristics allow potential use of gamma/delta cells as immunotherapy in combination treatment. For example, they can be used as a substrate for CAR T cell generation and particularly benefit from combination with RT.

#### Abscopal effects

Abscopal effects, referred to as the immune-mediated distant effects of RT, emerged as a significant observation when combining RT with immune-stimulating therapies or in disinhibiting immune suppression through interventions with ICIs [96, 97]. As abscopal responses were demonstrated in preclinical models, they were found to be mediated via T cells and were absent in immune-suppressed animals [96]. In this context, CAR T cell therapy may benefit from interaction with irradiation through similar, semi-similar or additional mechanisms as compared to ICIs-RT, e.g., via retargeting CAR T cell response towards non-irradiated tissues [97]. Such an interaction was demonstrated in a preclinical model of lung cancer in which mice were injected with lung adenocarcinoma cells simultaneously at several locations: pleura, chest wall, peritoneum, and flunk. Mesothelin-targeted CAR T cells were locally injected into a single tumor site, the pleura. In the RT group, the pleural tumor was irradiated with a single sub-therapeutic RT dose prior to CAR T cells injection. Locally-injected CAR T cells promoted the abscopal effect in distant tumor sites, and this effect was potentiated via RT [98].

CAR-T/RT abscopal-like responses were already documented also in a clinical setting. BCMA-targeted CAR T cells were combined with RT in a clinical trial for relapsed/refractory multiple myeloma (NCT03070327) [99]. The patient received CAR T cells after preconditioning with chemotherapy and developed neurological and cognitive symptoms after CAR T injection. These side effects were treated with high dose steroids and 20 Gy fractionated RT to brain and spinal cord given from the 6th day after CAR T cell therapy and lasted two weeks [99]. The patient developed cytokine release syndrome 3 weeks after CAR T cells’ injection, which coincided with the end of the last RT fraction. Moreover, T cell repertoire expanded after RT, indicating activation of both CAR T cells and patient’s unmodified T cells [99].

RT can trigger not only T-cell mediated but also macrophage-dependent abscopal responses. In a study of small cell lung cancer, RT was combined with the anti-CD47 (‘do not eat me’) molecule therapy and initiated macrophage-mediated antitumor immunity [100].

Although the abscopal effects in RT and CAR T combinations are yet to be evaluated in clinical studies, the existing data on combined RT and IC inhibition is ambiguous. Theleen et al. reported increased rate of abscopal responses in metastatic NSCLC treated with a combination of RT and the PD-1 blocker pembrolizumab [38]. In another study, PD-1 ICI nivolumab was combined with stereotactic body RT (SBRT) for metastatic renal cancer in a phase 2 clinical trial with the intent to induce abscopal responses in distant metastases. However, the results were disappointing as no improved outcomes could be documented [101]. Similarly, no abscopal responses were observed in a phase 2 clinical trial combining nivolumab with SBRT for head and neck squamous cell carcinoma [102].

**Table 2:** Impact of RT on challenges of CAR T cell-based therapy. CAR T cell-based therapy faces complex challenges that can be overcome (*second column*) or aggravated (*third column*) by RT-mediated mechanisms

Challenges of CAR T therapy	Advantages of RT combination	Potential drawbacks of combination with RT
Limited CAR T cell trafficking	Improved penetration and infiltration. Upregulation of adhesion molecules (ICAM1, VCAM1), chemokines [83].	High-dose RT causes vascular disfunction and may impair infiltration [103].
Hostile metabolic conditions		RT-induced cell death mediates release of cellular ‘junk’, purines, ATP, decreases pH [71, 104].
Impaired vascularization, hypoxia	Low-dose RT normalizes vascularization, ameliorates hypoxia [71].	Ablative RT doses may induce endothelial cell death and may aggravate hypoxia.
Immunosuppressive microenvironment	Induction of immune stimulatory receptors CXCR3 and CCR5 and ligands CXCL-9, CXCL-10, CXCL-16 [87].	RT promotes infiltration with Tregs, shifts balance towards Tregs, as they are more radioresistant [105]. RT induces chemokine stromal-derived factor 1α (SDF-1α) that attracts infiltration with myeloid-derived suppressor cells [106].
T cell exhaustion	Increased expression of MHC class I and II, FAS and FAS ligand, TRAIL [106–108].	RT induces upregulation of immune checkpoint receptors and ligands [105, 109].
Tumor heterogeneity	RT can stimulate target antigen expression and mediate cell death on antigen-negative cells via other mechanisms (i.g. TRAIL) [88].	RT induces tumor mutations, mesenchymal phenotype, invasion, and may contribute to survival of more aggressive clones [110, 111].
On-target, off tumor toxicities	RT may improve on-target killing via improved trafficking and boosting target antigen expression [86, 89], 90.	

### Examples of RT/CAR T cells combinations in specific cancers

#### Breast cancer

RT is one of the key treatment modules for non-metastatic breast cancer and is administered within several months after surgery [112]. Whereas RT is employed to prevent locoregional recurrence, systemic therapy mainly controls metastatic spread. When metastatic spread occurs, RT is used again as a treatment option to ablate oligometastatic sites, to improve neurological status, and to reduce pain [113]. In breast cancer, CAR T cell therapy is evaluated solely in clinical trials targeting recurrent and refractory disease [114, 115].

One of the successful preclinical therapeutic approaches for triple-negative breast cancer is the use of a natural killer group 2D receptor (NKG2D)-ligand-targeted CAR construct [116]. This treatment can potentially benefit from neoadjuvant RT for the induction of the target ligand expression as NKG2D ligands are upregulated 24 h after 20 Gy RT [117]. Another promising CAR T cell target is Mucin1 (MUC1), an aberrantly glycosylated protein expressed in the majority of breast cancer cases. In preclinical models, MUC1-targeted CAR T cell therapy was antagonized by inhibitory TME and by upregulation of PD-1 and TIM-3 [118]. Anti-MUC1 allogeneic CAR-T cells are in phase 1 clinical trial for various solid tumors including breast cancer (NCT05239143).

The hepatocyte growth factor receptor MET is aberrantly expressed in breast cancer and is associated with poor prognosis [119]. When tested in phase 0 clinical trial (NCT01837602), the MET-targeted CAR T cells’ intratumoral injections had a tolerable safety profile for metastatic breast cancer. Cells were injected into cutaneous or lymph node metastases and resulted in MET-negative injection site biopsies in two evaluable patients [120]. In parallel, it was shown in various cancers including breast cancer that MET targeting acts synergistically with RT as these approaches target different tumor niches [121–124]. In vivo experiments combining RT with epidermal growth factor receptor (EGFR)-targeted CAR T cells in triple-negative breast cancer demonstrated synergistic interaction of both modalities mediated via NF-kB-ICAM1 activation [125].

To date, no combination of RT and CAR T for breast cancer has been reported in clinical settings.

#### Hematological malignancies

Hematological malignancies, while being mainly treated by systemic therapy, are sensitive to RT when used as an adjuvant, consolidation treatment option. For some specific malignancies, such as the MALT-lymphoma, RT can be used as a primary therapy [126]. The first clinical advances in CAR T cell therapy were reported for B-cell malignancies and targeting of CD19, an antigen expressed mostly on B cells, which greatly reduces the probability of off-target effects. Evidence also shows that RT can be implemented as a bridging strategy for CD-19-targeted CAR T cell therapy in Diffuse Large B-Cell Lymphoma (DLBCL) as a safe and effective option [127].

Along with CD19-targeted CAR T cells, other targets such as CD20, CD22, CD38, CD30, CD70 are under investigation in clinical trials for hematological malignancies [128] and interestingly, RT seems to have an upregulating effect for some of them. For example, CD20 expression in the Burkitt’s lymphoma cell lines Daudi and Raji doubles within one day after 0.5-2.0 Gy of RT [65]. The effect seems to be dose-dependent in the RT range of up to 10 Gy, peaks at 24 h and disappears by 48 h post RT [66].

Gene expression profiling of Molt-4 lymphoma cells following RT demonstrated induction of immunostimulatory genes including CAR T cell-activating genes such as CD70, OX40 ligand and Fas receptor [129]. In addition, expression of the allergy-associated B-cell differentiation molecule CD23 increased 24 h after 5-20 Gy RT in Ramos B-cell lymphoma cell line, with NF-kB starting to increase 1 h post-RT and contributing to inflammation response and CD23 induction [130].

Among the limited clinical evidence of advantages of CAR T cell-based therapy along with RT in hematological malignancies is a case report of a patient with refractory DLBCL who benefited from 5*4Gy fractions of RT prior to CD19 CAR T cell therapy given for largely CD19-negative disease. This case report suggests that such RT/CAR T cells combination can be successful when RT is given in a neoadjuvant setting [88].

In addition, B-cell maturation antigen (BCMA) CAR T cell therapy was promising when combined with neoadjuvant RT within the clinical trial NCT02546167 [131]. RT was a successful bridging strategy for CAR T cell therapy of Non-Hodgkin lymphoma, with an excellent local tumor control [132].

There are other emerging clinical trials testing the combination of RT with CAR T cell therapy for hematological malignancies where RT is tested prior to CAR T cell treatment for feasibility and safety at early stages [133]. These studies will hopefully give more definitive insights concerning potential clinical use of such combinations.

#### Prostate cancer

RT is an established treatment modality for prostate cancer in all stages of the disease [134], whereas CAR T cell therapy is mainly implemented in clinical trials for patients with recurrent disease. Target antigens for prostate cancer CAR T cell therapy include prostate-specific antigen, prostate acid phosphatase, prostate-specific membrane antigen (PSMA), prostate stem cell antigen (PSCA), and epithelial cell adhesion molecule (EpCAM) [135, 136].

Preclinically, CD276-targeted CAR T cells for prostate cancer had synergistic outcomes when combined with irradiation in vitro and in vivo [137]. Those effects were mediated by upregulation of CD276 on RT-resistant prostate cancer stem cells.

RT can have a dual role in conditioning T cell-based immunity for prostate cancer treatment. PSCA mRNA expression levels can be downregulated in post-RT patients’ biopsies [138], thus questioning the benefit of a combined RT and anti-PSCA treatment. Another inhibitory effect of RT in prostate cancer is related to the activation of macrophage colony-stimulating factor 1 (CSF1). CSF1 promotes migration of tumor-infiltrating myeloid cells (TIMs), and was found to be elevated in prostate cancer patients receiving RT. In vitro, CSF1 is upregulated 48 h after 3 Gy RT treatment and its blocking results in lower recruitment of TIMs and delayed tumor regrowth after RT in vivo [139]. Immune-activating role of RT was evident also in the NCT01303705 phase I clinical trial of castration-resistant prostate cancer where the treatment groups received 8 Gy RT prior to combination with immune activating OX40 (CD134) stimulation with different doses of cyclophosphamide. An increase in CD4 + and CD8 + populations was observed irrespectively of cyclophosphamide dosage, along with an increase in IFG and interleukin-2 (IL-2) secretion [140]. As RT may exhibit a dual role on T cell activation in prostate cancer microenvironment, more research is needed to determine the best combination protocol.

#### Glioblastoma (GBM)

RT is along with surgery and chemotherapy one of three standard treatment modalities for GBM [141]. RT is also one of the few therapeutic options after inevitable tumor recurrence where no standard treatment is available․ These patients are being enrolled in various clinical trials that nowadays also include CAR T cell-based interventions. Recent CAR T cell trials showed potential promise but at the same time plausible risks of the use of CAR T cells for GBM treatment [142]. So far, interleukin 13 receptor subunit alpha 2 (IL-13R-alpha2), epidermal growth factor receptor variant III (EGFRvIII), human epidermal growth factor receptor 2 (HER2), CD276, Diganglioside GD2 (GD2), Ephrin A2 (EpA2) and PD-L1 [143, 144] have been exploited for the use in GBM treatment as all of them are frequently expressed in this malignancy.

Within the framework of RT and CAR T cell treatment combination, various RT-related effects on GBM cancer cells were documented, including induction of tumor antigens, neoantigens, activation of non-specific immune responses, and overexpression of immune checkpoint molecules [86, 89, 145]. NKG2D-targeted CAR T cells employed together with RT showed a synergistic effect in a murine glioma model [89]. The underlying mechanisms involve immune activation in both tumor cells and in brain tissue. As shown by Ivanov and Hei, a single dose of 10 Gy resulted in a dramatic increase in Il-6, Il-8 and TGF-beta expression levels in the U87MG cells 24 h post-irradiation. At the same time, surface expression of the Fas ligand increased markedly whereas a moderate increase in TNF-related apoptosis-inducing ligand (TRAIL) receptor 2 expression and in TRAIL production was observed [146]. In parallel, 35 Gy of RT induces brain edema and increases permeability of brain vessels via cyclooxygenase-2 (COX-2) activation within 4-24 h after RT, which can affect T cell infiltration [147]. Besides, TNF-alpha is produced by different cell types, including macrophages and tumor cells, and its production peaks 4-8 h post-RT [148]. These processes can favor early injection of CAR T cells within the course of a standard RT regimen for GBM.

Opening of the blood-brain-barrier (BBB) can also be one of the important mechanisms to induce immune response. Already 7 Gy of RT opens the BBB, and this effect is preserved for up to several days. This RT-induced BBB opening along with a massive cytokine release by both tumor and non-tumor cells can attract immune cells into the lesion. It was shown that 25 Gy RT increases activation of inflammatory transcription factors, production of interleukins IL-la, IL-1, IL-2, IL-3, IL-4, IL-6, IFN-gamma, and tumor necrosis factors. Most cytokines increase within hours after RT and decrease after 24 h [149]. It is important to highlight that IL-1 and TNF contribute the most to acute RT-induced inflammation [150], and this acute inflammation is suppressed by dexamethasone, which is commonly prescribed to GBM patients and can disturb potential benefits of RT for CAR T cell-based therapies [151].

In addition to the acute RT effects described above, a lasting and durable influence of RT on the immune profile of the brain was documented. In a longitudinal study, which studied the morphology of mouse brains irradiated with single RT doses of up to 35 Gy, ICAM-1, CC-chemokine ligand 2 (CCL2) and TNF-alpha protein expression increased during the first days, whereas CD3 + and major histocompatibility complex II positive (MHCII+) cell counts started to increase in the group that received the dose of 35 Gy in the first month and were maintained for up to one year [150, 152].

These mechanisms suggest that satisfactory results could be achieved in GBM when CAR T cell treatments would be combined with neoadjuvant RT.

#### Gastrointestinal (GI) tumors

CAR T cell therapy is being tested in various GI malignancies, most of which typically share common targets [153].

In esophageal malignancies, antigens such as EpCAM, HER2, and Muc1 are predominantly studied as CAR T cells’ targets [153]. Although neoadjuvant RT for esophageal cancer is currently used with chemotherapy for locally advanced disease [154–156], there is little preclinical and clinical evidence for possible RT and CAR T cell therapy combinations in this cancer type. A decrease in HER2 expression observed following chemoradiotherapy could for example render a combined treatment by RT and anti-HER2 CAR T cell-based therapy ineffective due to antigen loss [157].

In gastric cancer, RT is one of the key treatment options due to considerable advances in precision organ targeting and the development of intraoperative modalities that led to high RT effectivity [158]. As reported in a meta-analysis of randomized controlled trials, adjuvant RT improves overall survival in all patient subgroups by about 20% [159]. The few available CAR T cell-based studies in gastric cancer suggest folate 1 receptor (FOLR1) and NKG2D as relevant targets [160]. NKG2D upregulation on epithelial cells upon 10 Gy in vitro RT within the first day indirectly indicates potential benefit of concurrent RT and NKG2D CAR T cell-mediated targeting [161, 162].

For colorectal cancer, RT is implemented in all stages of the disease and can be scheduled before, during, or after surgery [163–165]. A variety of antigens present on the surface of colorectal cancer cells open wide opportunities for CAR T cell-based treatment. These antigens include carcinoembryonic antigen (CEA), MUC1, MET, EpCAM, HER2, EGFR, PD-L1, NKG2D, CD133, epithelial glycoprotein 2, epithelial glycoprotein 40 or the tumor-associated glycoprotein 72 [153]. Increased expression of some of these antigens following RT documented in some studies favors the combination of RT with CAR T cell-targeted treatment. For instance, a single dose of 6 Gy doubled MUC1 expression in HT-29 colon cancer cells, the increase starting from 24 h after RT and reaching a plateau on day 4 [67]. Similarly, 5-15 Gy irradiation increased CEA expression in the MKN45 gastric adenocarcinoma cell line after four days in a dose-dependent manner [68]. In accordance with this data, Garnett et al. later demonstrated increased killing of CEA-expressing colon cancer cell lines three days after 10 and 20 Gy RT doses via a CD8-T-cell-dependent mechanism. This study investigated a panel of colon cancer cell lines and identified a tendency to increase the expression of Fas, ICAM-1 and MHC-I after irradiation. Increased expression of those molecules may mediate improved cellular immune response [166]. Similarly, in a murine colon adenocarcinoma model, irradiation induced upregulation of the Fas ligand and ICAM-1 in a dose-dependent manner. Fas upregulation was stable within at least four days and four generations of cell division. As a result, those effects mediated an improved response to anti-CEA adoptive T cell therapy [167]. In addition, concurrent use of RT with a bispecific antibody targeting CEA improved antitumor response in vivo [168].

Other potential CAR T cell targets for combination with RT in colorectal cancer are PD-L1 or radiation-induced neoantigens. An increase in PD-L1 expression in CT26 murine colon carcinoma cells implanted in mice was observed within one week after receiving RT in a 5*2Gy regimen [69]. However, the RT and anti-PD-L1 therapy combination requires a well-determined timing as the comparison of three different schedules suggested that the first week of RT would be optimal [69]. In HT-29 human colon adenocarcinoma cell line, 1*8Gy and 4*2Gy radiation doses upregulated karyopherin alpha 2 (KPNA2) expression during the first three days after the last dose [64]. In an in vitro study, KPNA2 activated cellular immune response, highlighting the pleiotropic role of RT in combination with immune-mediated therapies [64]. Another RT-induced neoantigen is the extra domain B (ED-B) of fibronectin, utilized as a target for L19. In the C51 mouse colon carcinoma model, 10 Gy radiation combined with immunocytokine L19-Il-2 prolonged animal survival and resulted in 75% long-term survivors compared to no surviving animals following each of these modalities as monotherapy. The effect was T-cell mediated and dependent on RT-IMT combination timing, favoring concurrent combination within the same week [169].

#### Pancreatic cancer

Pancreatic cancer, one of the most aggressive cancers with poor prognosis, is being widely studied to find new treatment options. RT is used in various settings including external beam RT, stereotactic body RT and brachytherapy, along with clinical trials investigating novel regimens [170–172]. CAR T cell therapy targeting HER2, MUC1, CD24, PSCA, NKG2D and CD47 was evaluated in preclinical and clinical studies [173, 174].

Interestingly, a particular combination of CAR T cell-based therapy and irradiation in pancreatic cancer was studied by DeSelm et al., where irradiation augmented CAR T cell therapy outcomes via an effect on antigen-negative cells. Neoadjuvant 2 Gy irradiation potentiated sialyl Lewis-A (sLeA)-targeted CAR T cells in killing not only sLeA-positive but also sLeA-negative cancer cells. CAR T cells initially interacted with antigen-positive cells, triggering the expression of TRAIL and further neutralized antigen-negative cancer-cells via a TRAIL-dependent mechanism [88]. As this mechanism may play a role in other cancer types as well, these findings suggest an attractive way to overcome CAR T cell therapy-related antigen-negative recurrence.

Considering immune escape of immunologically ‘cold’ pancreatic cancer, RT was studied as a means to attract immune cells. However, although RT increased tumor infiltration by CD8 + T cells in murine pancreatic ductal adenocarcinoma cell lines, an increase in PD-L1 expression 24 h after RT has been observed, which may induce exhaustion of the infiltrating lymphocytes [175].

Nevertheless, the growing interest in pancreatic cancer IMT should stimulate further investigations on the potential of RT in overcoming immune indifference of this cancer to eventually become a successful combination strategy.

#### Melanoma

RT and IMT based on ICIs are standard treatment options for melanoma, an immune-dependent tumor [176, 177]. MET-, CD70-, HER2-, GD2- as well as vascular endothelial growth factor receptor (VEGFR)-targeted CAR T cell therapies were already investigated as potential melanoma treatment strategies [178, 179].

In RT and CAR T cell therapy combination settings, RT can be beneficial for the induction of immune response, antigen expression, presentation to APC, and improved infiltration of immune cells. For instance, anti-VEGFR therapy can be potentiated by antigen upregulation in a dose-dependent manner as documented in canine oral melanoma cell line TLM 1 within 6 days after 2-10 Gy irradiation [180]. Irradiation can also activate T cell response of otherwise non-immunogenic and aggressive melanoma, such as the B16 cells [181]. The immune response seems to depend on a particular RT treatment schedule, the 20 Gy*1 dose being superior over the 4*5 Gy regimen. This RT-mediated immune response was absent when T cells were depleted. Moreover, these cells became radioresistant in case of T cell depletion. The superiority of the ablative single dose of 20 Gy over the 4*5 Gy regimen is possibly related to the death of migrated lymphocytes with sequential doses of RT in the 4*5 Gy scenarios. It is important to note that chemotherapy may interfere with the immune-activating role of RT. B16 tumor-bearing mice receiving 3*15 Gy had less metastases compared to mice receiving RT with chemotherapy [181]. The advantage of ablative single dose regimen for immune induction was confirmed in ovalbumin-expressing B16-F0 melanoma-bearing mice. When animals received a single dose of 15 Gy or a fractionated 5*3 Gy RT, both regimens markedly decreased tumor growth. However, 1*15 Gy was superior in increasing APC in lymph nodes and tumor infiltration of CD45 + cells [182].

To summarize, melanoma as an immune-dependent tumor responds well to immune-mediated therapies, and the treatment benefits from RT. RT is more potent when used in a neoadjuvant setting for induction of cellular response, and can help to overcome challenges of aggressive and poorly immunologic melanomas.

#### Lung cancer and mesothelioma

RT is one of the key options for lung cancer treatment at all stages of the disease and is also widely studied due to its immune-activating capacity, especially following high-dose fractionation [183]. Considering the poor prognosis and the high mortality rates of lung cancer and mesothelioma, effects of many novel therapies including CAR T cell-based treatments are extensively investigated in both preclinical and clinical settings. With respect to suitable CAR T cell targets, numerous antigens including CEA, MUC1, HER2, EGFR, mesothelin, fibroblast activation protein, GD2 as well as receptor tyrosine kinase orphan-like receptor 1 have already been assessed [184–186].

In mice, lung RT was shown to induce expression of multiple cytokine genes, including TNF-alpha, IL-1alpha, IL-1beta, IL-2, IL-3, IL-4, IL-5, IL-6 and IFN-gamma, with a peak observed 4 h and a decrease noted 24 h post-RT, with IL-1alpha and TNF being the two major players in the RT-induced-inflammatory response. Although the effect does not last long after a single dose, it can be sustained by employing a fractionated RT regimen [187].

The limited clinical data on the combination of CAR T cells with RT suggest that the overall immune-activating effect of RT can be beneficial for patients and works efficiently in combination with ICIs. In one of the successful trials, RT improved patient outcome when given prior to anti-CTLA-4 antibody ipilimumab for metastatic lung cancer [188]. Combination of RT (5*6 Gy or 3*9.5 Gy) and ipilimumab induced systemic immune response in 7 out of 21 patients. Blood analysis of the responders revealed an increase in interferon-β as well as in the repertoire of expanded T cells with antitumor immunity [188].

In addition, retrospective analysis of the KEYNOTE-001 trial that included patients who received the PD1-blocking antibody pembrolizumab prior to RT showed that these patients had a significantly longer progression-free and overall survival as compared to patients treated by RT only [189].

In summary, promising opportunities seem to exist for combinations of high-dose RT for induction of immunity and infiltration of T cells in lung cancer and mesothelioma, especially when the treatment is optimized by the use of checkpoint inhibitors.

## Challenges and risks for the combination therapy

### Impact of RT/CAR T cell combined treatment on TME

Despite showing promises in improving tumor response to CAR T cell-based approaches, RT may be able to solve only some difficulties that the CAR T cell therapy faces. At the same time, RT also has an impact on TME cells that can be deteriorating for CAR T cells. RT can for instance impact the balance of T effectors/Tregs in favor of Tregs that are immunosuppressive by nature and more resistant to RT. It was shown that RT increased CD4 + CD25 + Foxp3 + Treg population in a mouse prostate cancer model [190] and a dose-dependent Tregs increase was also associated with RT doses higher than 7.5 Gy in a mouse melanoma model [191]. These results are in line with a clinical study involving patients with localized prostate cancer who received 65.6 Gy carbon ion RT. Tregs frequency increased after irradiation, although their proliferative capacity did not change [192]. Local irradiation activated chemokine stromal-derived factor 1α (SDF-1α) and recruited myeloid precursor cells, contributing to tumor progression in murine lung cancer model. This effect was suppressed when bone marrow was irradiated by whole body irradiation, and partially restored with infusion of bone marrow-derived cells [193]. RT boosts IFN-gamma release in TME, and activates cGAS-STING pathway, contributing to the increase of IC PD-L1 expression [194, 195]. Moreover, RT contributes to tumor cell death, promoting the release of metabolic intermediates, peptides, ATP, and purines that decrease pH and exhaust T cells [196]. When combining RT with CAR T cell therapy, RT-mediated IC modulation should be evaluated and optimal timing, dose and addition of IC blocking agents should be considered. How RT influences various ICs and what are the therapeutic implications is further discussed.

Despite the promise of a synergistic combination, employing two treatments such as RT together with CAR T cell therapy yields some challenges. As testing of these combined regimens in clinical trials started only recently, it is difficult to predict an optimal treatment scenario regarding the timing, dose, and sequence of the two treatment modalities [197].

### Side effects and toxicities

One of the limited evidences on RT and CAR T cell therapy combination in clinic demonstrated favorable safety profile when compared with chemotherapy and CAR T cell treatment combination. In phase 2 clinical trial for relapsed/refractory diffuse large B-cell lymphoma (DLBCL) (NCT03196830), 6 patients received CAR T cell-based therapy combined with RT, and 4 patients received CAR T cells in combination with chemotherapy. All patients in the RT group responded to the treatment with no severe cytokine release syndrome. At the same time, the chemotherapy group had a lower response rate (25%), and all these patients experienced the cytokine release syndrome [198].

It is still unknown how RT will contribute to the risk of life-threatening toxicities of CAR T cell-based therapies such as cytokine release syndrome or macrophage activation syndrome and if those syndromes will be aggravated with RT addition [199]. New side effects and previously not described adverse events can be observed after such a combination; thus, wisely calculated combinations and implications from clinical research must be implemented [200, 201].

NCT04601831, a phase I/II trial, uses hypofractionated RT to sensitize relapsed/refractory non-Hodgkin lymphoma (R/R NHL) to CD-19-CAR T cell treatment [202]. Another early-stage trial, NCT05574114, is currently recruiting to test the safety of bridging 10*3 Gy RT with CAR T cell therapy for relapsed or refractory B-Cell lymphomas. In this study, patients will receive 9*3 Gy over the first 15 days of the trial. Further, they will receive lymphodepletion chemotherapy, and the last 3 Gy dose of RT will be given two days before CAR T cell infusion [203]. NCT05621096 is another interventional phase I trial of bridging RT for lymphomas. Patients will receive 2* 2 Gy one week before CAR T cell treatment and up to 32 Gy starting from one month after CAR T cell therapy [204]. NCT04888338, an observational clinical trial from the MD Anderson Cancer Center, has completed data collection to evaluate outcomes after radiation and CAR T cell-based therapy for various haematological neoplasms [205]․ All these studies will help to elucidate potential risks, side effects, and adverse events of RT/CAR T cell-based combination treatments and whether the synergistic effect regarding the treatment efficacy is not associated with an additive toxic effect. Potential optimism in this direction may be found in results of in vivo studies reporting the synergistic effect of these combination treatments without associated increase in toxicities, weight loss or changes in blood biomarkers [88, 89, 125].

### Optimal timing and dosing of RT and CAR T cell-based therapy

An optimal timing and dose scheme for the RT and CAR T cell combination therapy is an open question since so far only a few studies accentuated those variables. Weiss et al. used a single dose of 5 Gy in combination with 3 doses of CAR T cells administered two days before RT, on the day of RT, and two days after the RT treatment [89]. DeSelm et al. employed a single dose of 2 Gy and administered a single CAR T cell injection shortly after RT [88]. In addition, Zhou et al. compared various regimens of RT and CAR T cell therapy combination in their studies and show that a single dose of 10 Gy was as tolerable as 3 Gy in 3 fractions, and administration of CAR T cells 4 h after RT had better outcomes than the later administration at 48 h [125].

The uncertainty in timing and sequence of RT and CAR T cell-based combinations is clearly demonstrated also in the designs of ongoing clinical trials where the treatment schemes vary significantly. The NCT04601831 trial aims to ‘re-prime’ the tumor after CAR T cell therapy by irradiating metabolically active lesions 30 days after CAR T cells’ injection [202] whereas the NCT05574114 phase 1 clinical trial, where RT is used in a ‘bridging’ scheme for chemotherapy, the last fraction of RT is given two days before CAR T cells’ injection [204]. In the phase 1 trial from the University of Nebraska (NCT05621096), the patient receives low-dose RT up to one week before and a clinically relevant RT dose one month after CAR T cells’ injection [204] and another early phase clinical trial from University College London (NCT04726787) combines clinical doses to bulk tumors with low-dose RT to other areas given 2–3 weeks before CAR T cells’ injection [206].

## Conclusions

CAR T cell-based treatment is a powerful immunotherapy in hematological tumors. However, clinical responses to CAR T cell therapy in cancer patients with solid tumors are so far limited. Here we cover promising observations seen through the combination of ICIs/RT largely in the form of abscopal effects to adapt combinations with RT to overcome the current obstacles that compromise cytotoxic activity of CAR T cells for effective eradication of solid tumors. RT is one of the main cancer treatment modalities with proven local and systemic anti-tumoral effects. Obviously, the abscopal effects seen in clinical setting with ICIs/RT combinations are quite limited and do not represent a general phenomenon. The understanding of why only few patients develop abscopal effects in such setting need to be investigated using precision oncology tools. In the next step, those approaches will need to be similarly applied for CAR T cell/RT combinations. Additionally, there is both direct and indirect evidence that RT may overcome some of the current limitations of CAR T cell therapy in solid tumors and may therefore serve as a powerful tool to improve CAR T cell function. However, prior to achieving this goal substantial efforts must be undertaken to optimize fractionation, dosing and timing with respect to the optimal modality of RT to be combined with CAR T cell therapy. Furthermore, an in-depth characterization of biomarkers and mechanisms that may drive such combinations is urgently needed to safely identify patients that will profit from the combination therapy and to minimize side effects.

## Data Availability

Not applicable.
